# Therapeutic Drug Monitoring and Outcome of Infliximab Therapy in Pediatric Onset Inflammatory Bowel Disease

**DOI:** 10.3389/fped.2020.623689

**Published:** 2021-01-13

**Authors:** Kaija-Leena Kolho

**Affiliations:** ^1^Children's Hospital, Helsinki University Hospital and University of Helsinki, Helsinki, Finland; ^2^Faculty of Medicine and Medical Technology, Tampere University, Tampere, Finland

**Keywords:** anti-TNF, children, Crohn disease, ulcerative colitis, unclassified colitis

## Abstract

Inflammatory bowel disease (IBD) with pediatric onset has become more prevalent during past decades. Thus, the number of patients with moderate to severe disease subtype treated with antagonists to tumor necrosis factor alpha (TNFα) has concurrently risen. Most pediatric patients initially respond to these drugs but will need dose escalation during the first year of therapy. As pediatric data regarding therapeutic drug monitoring during therapy with TNFα-blocker adalimumab are sparse, this review focuses on the literature on therapeutic drug monitoring of infliximab and how it may guide management.

## Introduction

Infliximab, which is a monoclonal antibody to tumor necrosis factor alpha (TNFα), has become the mainstay therapeutic agent for treating moderate to severe pediatric inflammatory bowel disease (PIBD). This is comparable with the management of adult IBD. The patent for infliximab expired in Europe in 2015 and nowadays there are on the market several biosimilars to the original drug Remicade (Janssen Biologics) (1, 2). So far, there are no safety alarms related to the use of biosimilars and the efficacy in clinical practice is comparable to the original drug (2). The access to the different drugs and their use varies between countries. Infliximab and its biosimilars are licensed for use in children who are at least 6 years of age and have moderate to severe Crohn disease or ulcerative colitis. Several countries, such as Finland, allow off-label use in younger children at the physicians' discretion. Induction phase of the drug covers 8 weeks and the three first doses are administered intravenously at week 0, 2, and 6, and mostly as 5 mg/kg. Maintenance phase and the fourth dose follow after 8 weeks (1–5). Most patients with PIBD, especially with Crohn disease, initially respond to infliximab therapy but will need dose escalation during the first year of therapy (6–8). This includes shortening of the interval and increasing the dose. To guide therapeutic decisions, the use of trough level measurementsof infliximab have emerged (9–12). In PIBD, the published reports on drug level monitoring mostly concern the use of the original drug and the use of biosimilars is less addressed. This review discusses use of infliximab trough level measurements in the management of PIBD.

### Why Do Patients With Pediatric Onset Inflammatory Bowel Disease Treated With Infliximab Need Trough Level Measurements of the Drug?

Induction of infliximab therapy results in responders in rapid improvement of clinical symptoms, blood and fecal inflammatory markers (13, 14), and even weight gain (15, 16). For example, the normalization of fecal calprotectin levels may occur already during the first 2 weeks of the therapy when response is complete (17). Perhaps unexpectedly, in partial responders the improvement in the objective markers of inflammation is only modest between therapy weeks 2 and 6 (17–19). During the first year of therapy up to 80% of the pediatric patients may need therapy escalation and one third may eventually be judged as non-responders and need switching to other therapeutic drugs (6–8, 20). When non-response is timely detected and therapy adjusted accordingly, the therapeutic drug monitoring improves cost-effectiveness of the management (21, 22).

Most reports on infliximab trough levels in PIBD originate from single centers and are retrospective reports. Therefore, the number of included patients is limited, the timing of trough level measurements is variable, and in most studies, only samples taken during maintenance therapy are included. Table 1 summarizes some of the most recent studies with considerable number of patients with PIBD (23–31). The general conclusion from these studies is that in the group of patients with clinical remission or mild disease activity, the trough levels of infliximab are statistically significantly higher than in the group of patients with poor or unsatisfactory response. Also, the proportions of patients with undetectable or low levels of the drug are higher in the latter groups. However, the absolute values of the median (or mean) values are close to one another and there is significant overlap with the interquartile ranges of the values. In current clinical practice the target range is somewhat higher than in the early days of adopting the infliximab therapy in PIBD. There is no clear consensus of the target trough levels but levels above 3–5 mg/mL are considered adequate (11). This applies to all disease subtypes, i.e., Crohn disease, ulcerative colitis and unclassified colitis (31–33). However, in acute severe colitis to reach these levels may need vigorous dosing probably due to increased clearance of the drug in active inflammation (3, 5). Notably, in perianal fistulazing disease the target level for best prediction of fistula healing may be as high as 12.7 mg/mL (34). Importantly, higher trough levels do not result in increased risk for infections or other adverse events (35). It is remarked that there is variability in the performance of the different assays for measurement of the drug levels hampering the comparison of absolute values in different settings (36). There are less data on the performance of the commercial assays when using biosimilars but according to our experience the results are comparable with infliximab and its biosimilars (20).

**Table 1:** Summary of the main studies included in the review.

**References**	**No. of patients**	**Age years**	**Trough level measurements**	**Drug antibody measurements**	**Results from trough level measurements**
Naviglio et al. (23)	49 Crohn 34 UC 15	14 median (IQR 11.6-16.2)	*n* = not available at week 6,14,22,54	When IFX level <1.5 mg/mL 10 patients (20.4%) had low levels and were positive for drug antibodies	**at week 6** In clinical remission median 9.8 μg/mL (IQR 8.4 to 12.6) vs. 7.1 μg/mL (IQR 4.7 to 9.8 μg/mL) in active disease **at week 54** In clinical remission median 6.1 μg/mL (IQR 3.8-9.6) vs. 1.4 μg/mL (IQR 0.35-2.8 μg/mL) in active disease
Lega et al. (24)	83 Crohn 76 UC 7	14 Median (IQR 11-16)	*n* = 243 during maintenance	25 (30%) positive for drug antibodies	In proactive monitoring group median 9.1 μg/mL (IQR 6.2-13) vs. 5.4 μg/mL (IQR 1.7-10.8) in the group of standard care
Clarkston et al. (25)	72	13 mean (SD±4)	*n* = not available during induction	None	In clinical responders median 27.8 μg/ml (IQR19.5– 40) at infusion 2 and 14 μg/ml (IQR 8.3–24) at infusion 3
van Hoeve et al. (26)	35 Crohn 23 UC 12	12.7 (IQR 10.2–14.6)	*n* = 35 at week 12 to 14	In 3 with undetectable trough levels but all negative	Clinical 4.6mg/mL (IQR2.7) vs. 1.5 mg/mL (IQR 0.9–3.0), biological 4.6 mg/mL (IQR 2.5–10.3) vs. 2.6 mg/mL (IQR 0.3–3.2) and combined clinical/biological remission 6.0mg/mL (IQR 3.2–12.0) vs. 2.6mg/mL (IQR 1.1–3.2) at week 52 compared to children not meeting these criteria (all *P* < 0.002)
van Hoeve et al. (27)	52 Crohn 33 UC 19		*n* = 686 during maintenance		In clinical, biological and endoscopic remission median levels were 5.2, 5.7 and 6.5 μg/mL, respectively, and higher than in those not meeting these criteria 4.2 μg/mL (IQR 2.6-6.7) 3.7 mg/L (IIQR 1.8-5.4) and 1.2 mg/L (IQR 0.03-4.4; *P* = 0.01), respectively
Ungar et al. (28)	63 Crohn 50 UC 13	median 14 (IQR 11.75-16)	*n* = 773 during induction and maintenance (*n* = 682)	At week 6 and 14 median levels of drug antibodies higher in patients discontinuing the therapy during 1 year vs. patients with ongoing therapy	In clinical remission 4.0 mg/mL (IQR 2.0–6.4) vs. 2.25 mg/mL (IQR 0.5-4.7; p < 0.0001) in clinically active disease Median levels **at week 2** higher when in clinical remission at week 14: 12.8 mg/mL (IQR 9.7–16.2) in clinical remission compared to 7.6 mg/mL (IQR 2.1–12.9) in clinically active disease (*P* < 0.02) Median levels **at week 6** higher when in clinical remission at week 14: 8.4 mg/mL, (IQR 6.9–17.0) in clinical remission vs. 5.5 mg/mL (IQR 0.27–12.3) in clinically active disease (P < 0.04)
Merras-Salmio and Kolho (19)	145 Crohn 101 UC 32 IBDU 12	14.8 median (IQR 12.5–16.0)	*n* = 475 during induction and maintenance	208 analyses when IFX <2.0 mg/L, 65% positive when IFX <0.2 mg/L, 89% positive	In remission and/or ongoing therapy median 3.7 mg/L (IQR 1.8-5.4) vs. 1.2 mg/L (IQR 0.05-4.4) in the group with loss of efficacy or no efficacy
Choi et al. (29)	39 Crohn 29 UC 10	<19	*n* = 99 during maintenance	Drug antibodies 0/16 with good response and 7/23 with poor response	In clinical remission levels higher than in those with poor response median 3.99 μg/mL (IQR, 0.30-21.96) vs. 0.88 μg/mL (IQR, 0.00-6.80, p=0.002)
Rolandsdotter. (30)	45 Crohn 32 UC 13	7-18	*n* = 93 during maintenance	12 samples with undetectable trough levels and all positive for drug antibodies	In clinical remission mean levels higher (7.2 μg/mL) compared to active disease 4.5 μg/mL (*p* < 0.05)
Deora et al. (31)	73 Crohn 52 UC 18 IBDU 3	median 16.12 (IQR 14.34-17.91)	*n* = 107 during maintenance	None	In UC median 4.2 μg/mL (IQR = 2.4–9.477) and in Crohn 5.9 (IQR = 3.9–12.65) in suboptimal response (*n* = 38; 35.5%) median 1.8 μg/mL (range 0.04–3.4)

*IBDU, unclassified inflammatory bowel disease; IQR, interquartile range; UC, ulcerative colitis*.

In studies on drug monitoring in PIBD, the grouping of the patients according to their therapeutic response is most often based on clinical remission, defined with PCDAI or PUCAI indices (25, 30). In the study by van Hoeve et al. (27), 40 patients underwent endoscopy after a median of 12.6 months and there were in total 87 time points for endoscopic data. In 54% of the visits, the patients were in endoscopic remission. In those children who were in remission, the median infliximab trough levels were significantly higher compared to those not in remission (Table 1).

In clinical practice, most have measured trough levels when the therapeutic response is not optimal or there has occurred an adverse event that might be related to the presence of drug antibodies and low trough levels. Although drug antibody formation is rare during first weeks of therapy (18, 37), there has been discussion whether trough levels should be measured already during induction. Suboptimal trough levels may guide therapy enhancement and in theory, adjusting the dosing early may prevent loss of response. If trough levels are suboptimal at week 6 to 8, this may associate with inferior therapeutic response (10, 38) and increase the risk for antibody formation prior the first maintenance dose (fourth dose of the therapy). To avoid this, many clinics have started to monitor the trough level for the first time at week 6 to be able to enhance the therapy in due course when needed. Indeed, higher trough levels at the first dose of maintenance predicted sustained remission at 1 year (26). Recently, it was suggested that infliximab concentration of ≥29 μg/ml at infusion 2 and ≥18 μg/ml at infusion 3 was associated with improved outcomes (25) in infliximab-treated patients with PIBD.

When decisions on drug dosing are based on clinical data, inflammatory markers and drug levels, most pediatric patients need therapy adjustment compared to standard of care (39). One of the first studies reporting comparison of patients with PIBD followed up with proactive drug level monitoring and patients with standard care, concluded that there was a difference in the median values of the drug levels between these groups but no difference in the therapeutic outcomes (24). Thus, although in the current practice most aim to achieve higher trough levels to improve therapeutic response and to diminish loss of efficacy, the data on showing that this indeed results in better outcomes are sparse.

The mechanisms for the loss of response to infliximab therapy are not completely understood. Several hypotheses have been proposed including e.g., (1) drug antibody formation (2) a high inflammatory load of the disease and rapid turnover of the drug (3) development of an alternate inflammatory pathway. To avoid drug antibody formation, some favor combination therapy with immunosuppressants although this increases the risk of severe adverse events (40) and does not invariably protect from drug antibody formation (19). In young children, the clearance of infliximab has been estimated to be more rapid and to achieve target levels of the drug, higher doses around 10 mg/kg and shortening of the interval to 4–5 weeks may be needed (41). A recent study summarized data from 215 patients with PIBD treated with infliximab and with trough level measurements during induction and maintenance. Regarding patients younger than 10 years of age, two thirds of the patients had trough levels below the target level at the start of the maintenance therapy. After one year of therapy, the dose requirements and risk to develop drug antibodies were higher in the group of young patients compared to the older patient group (41). The therapeutic outcome in the young patients, however, was not different from the older patients (24, 41). However, it is not just weight or age of the patients that have an impact on the trough levels and the outcome of the therapy. The level of inflammation as reflected in the levels of albumin and inflammatory markers is a key player in drug pharmacokinetics (42). Therefore, in patients with high inflammatory load, higher dosing of infliximab is needed to reach target levels (3, 5). Disappointingly, a recent report stated that although trough levels during induction therapy with infliximab increased and antibody formation decreased, the proportion of pediatric patients maintaining their therapeutic response during the first year did not increase (20). It is not clear why some patients with trough levels considered as adequate, lose their therapeutic response. As this may occur after several months of maintenance therapy, it has been suggested that in these late non-responders the inflammatory pathway is altered and becomes resistant to TNFα blockade (43, 44). Indeed, the initial response to infliximab therapy during induction is predictive for the long-term outcome of the therapy both in children and in adults with IBD. Patients with low levels of fecal calprotectin at the end of induction are more likely to have a favorable long-term outcome compared to patients with elevated levels of fecal calprotectin reflecting ongoing inflammation after induction (7, 45).

### Is Determination of Drug Antibodies Necessary?

There are several different tests for the detection of drug antibodies, each having different levels of detection and cut-offs for a raised value. In most assays, the level of infliximab interferes with the test performance and the result is negative when there is measurable infliximab in the blood (32). When drug levels are undetectable, the risk of antibody formation is increased and thus, a rapid therapy escalation is needed. In clinical practice, this most often includes shortening of the interval and increasing the dose (e.g., (10, 41)). However, when trough levels of the drug are well within the target range, the impact of measurable drug antibodies is less likely to be of clinical importance. Notably, drug antibodies may appear already during induction (18, 46) although in most cases they are detected during maintenance at the time of secondary loss of response (32). In the single-center report by Cohen and co-authors, drug antibodies with a detection limit of 3.1 U/mL for infliximab were present in one fourth of the anti-TNFα treated patients (47). They concluded that therapy optimization rather than switching to another therapeutic agent is advisable with antibody level < 10 U/mL. In several reports, it has been suggested that high antibody titers carry a risk for antibody-related adverse events, e.g., serum sickness type antigen-antibody reaction, but the definition of such a high level of antibodies is inconclusive. The pharmacokinetics, pharmacodynamics, and immunogenicity of infliximab in PIBD was recently comprehensively reviewed (12). Taken together, there is no need to measure drug antibodies when trough levels are within the therapeutic range but when trough levels are undetectable, drug antibody measurement is recommended and if the antibody titer is exceedingly high, it is advisable to discontinue the drug therapy.

## Conclusions

Therapeutic drug monitoring of infliximab is here to stay for clinical decision-making. Patient age, weight and severity of the disease are important contributors to be taken into consideration when adjusting the therapy. Currently, trough levels above 3–5 mg/mL are considered adequate but in fistulazing disease target levels may be higher (~10 mg/mL or more). An active infliximab trough monitoring strategy should be implemented with automatic measurement of drug antibodies in low trough levels. With such practice, patients with incomplete therapeutic response will have timely escalation of the therapy and possibly less drug antibody formation. When trough levels are within the target range, the determination of drug antibodies does not have clinical significance. With low trough levels and detectable drug antibodies, therapy enhancement may result in disappearance of the drug antibodies. However, with high titers of drug antibodies (the absolute values depending on the assay used), there is a risk for adverse antigen-antibody reactions and therapy should be discontinued at physician's discretion. When efficacy is not sufficient and trough levels are high, such patients are candidates for switching to another therapeutic agent. It seems that proactive therapeutic drug monitoring does not remarkably increase the proportion of patients having a long-term response to infliximab therapy but it aids in the recognition of the drug non-responders and therefore has the potential to result in a more cost-effective therapy.

## Author Contributions

K-LK conceptualization, interpretation of data, and writing original draft.

## Conflict of Interest

The author declares that the research was conducted in the absence of any commercial or financial relationships that could be construed as a potential conflict of interest.
